# Nonlinear nexus between corruption and tourism arrivals: a global analysis

**DOI:** 10.1007/s00181-021-02193-2

**Published:** 2022-02-07

**Authors:** Krambia-Kapardis Maria, Stylianou Ioanna, Demetriou Salomi

**Affiliations:** 1 Cyprus University of Technology, Limassol, Cyprus; 2 University of Central Lancashire, Pyla, Cyprus

**Keywords:** Nonlinearities, Threshold regression, Corruption, Tourism demand, C59, D73, Z32, L83

## Abstract

The relationship between corruption and tourism has been sporadically examined over the years. According to the existing theory, there is an inverted U relationship which implies that tourism demand initially increases as corruption increases (greasing the wheels) and after a certain threshold level of corruption, tourism demand decreases (sanding the wheels). Empirical studies so far concentrated on capturing the nonlinear relationship, by applying a simple linear model and by including corruption as a quadratic term. In the current paper, the authors revisit the “greasing and sanding the wheels” hypothesis by applying an advanced econometric technique, the threshold regression model, which deals with a key element of model uncertainty, namely parameter heterogeneity. In particular, using a sample of 83 countries from 2001 to 2018, the authors firstly examine if there is a nonlinear relationship between corruption and tourism, and then, they estimate the threshold value of corruption. According to the results, the null hypothesis of a linear model against the alternative of a threshold model with two regimes is strongly rejected. Furthermore, while the effect of corruption on tourism is positive in the low corruption regime and negative in the high corruption regime, a heterogeneous relationship is also found between other politico-socio-economic variables and tourism demand in the low and high corruption regimes.

**Supplementary Information:**

The online version contains supplementary material available at 10.1007/s00181-021-02193-2.

## Introduction

The growth in tourism since the 1950s is a global phenomenon and so is the concern about corruption. Regarding tourism growth, international tourist arrivals increased from 25 million in 1950 to 1.4 billion in 2018 (WTO 2018, 2019). In fact, the United Nation World Tourism Organization (UNWTO)’s Tourism Highlights noted in 2018 that international tourism arrivals in 2017 experienced the highest growth of 7% since 2010, contributing 10.4% of global GDP. Also, growth in international tourist arrivals continued to outpace the economy in January 2020 before the COVID-19 pandemic that had first affected China and subsequently spread across the globe becoming a devastating pandemic. According to the UNTWO’s Barometer on 18 January 2020 for all regions, international tourist arrivals (overnight visitors) worldwide grew 4% in 2019 to reach 1.5 billion.

The unprecedented COVID-19 pandemic at the time of writing (June 2020) makes accurate growth predictions impossible. Suffice it here to say that, according to UNWTO (2020a, b)’s “Impact assessment of the COVID-19 outbreak on international tourism” on 27 March 2020, “Current scenarios point to declines of 58–78% in international tourist arrivals for the year, depending on the speed of the containment and the duration of travel restrictions and shutdown of borders, although the outlook remains highly uncertain (the scenarios are not forecasts and should not be interpreted as such)”.

Undoubtedly, tourism is one of the forces that drives economic growth (Chen and Ioannides 2020), alleviates poverty in developing economies, creates jobs and generates tax revenue (UNWTO 2015). However, no country is devoid of corruption which, as discussed below, is related to tourism in the form of an inverted U-shaped curve.

The present paper aims to contribute to the literature on the relationship between corruption and tourism demand in multiple ways. The first research question the paper addresses relates to the direct relationship between corruption and tourism arrivals considering the likelihood of a linear or a nonlinear effect. Should a nonlinear relationship exist, the second aim of the paper is to examine if the “greasing the wheels’’ and the “sanding the wheels’’ hypotheses are valid which imply that tourism demand initially increases as corruption increases (greasing the wheels) and after a certain threshold level of corruption, tourism demand decreases (sanding the wheels). Finally, the third aim of the paper is to examine not only the direct effect of corruption on tourism demand under a low and a high corruption regime but also the indirect effect of corruption through the impact of other politico-socio-economic variables on tourism demand within these corruption regimes, a question which had not been empirically addressed before.

According to the results, the null hypothesis of a linear model against the alternative of a nonlinear threshold model with two regimes is strongly rejected. Furthermore, the “greasing the wheels’’ and the “sanding the wheels’’ hypotheses are confirmed which imply that the effect of corruption on tourism demand is positive in the low corruption regime and negative in the high corruption regime. In addition, a heterogeneous relationship is also found between various other politico-socio-economic variables and tourism demand in the low and high corruption regimes.

The rest of the paper is organized as follows. Section 2 reviews the relevant literature, while Sect. 3 describes the aims of the project, the data, and the model used in the study. Section 4 reports and discusses the main empirical results, while Sect. 5 examines the robustness of the results. Finally, Sect. 6 discusses policy implications and concludes the paper.

## Literature review

### Corruption

Regarding *corruption*, Kaufman and Vicente (2011) challenged the traditional definition of corruption as the “abuse of public office for private gain” arguing that there is a need to distinguish between illegal corruption, legal corruption, and no-corruption. In attempting to disentangle illegal and legal corruption, the same authors proposed a theoretical model by defining corruption as a “collusive agreement” or connections between the elite. The same authors refer to private sector agents and politicians who are involved in an exchange of favours over time. Once legislation protecting corruption is enacted and enforced, then legal corruption is created. This is known as “state capture”, i.e. passing legislation that favours few and selected individuals or organizations.

In addition to the private sector and politicians partaking in state capture, public officials may also create opportunities for bribes due to their discretionary powers (Swaleheen 2011). Thus, public officers act as independent monopolists (Shleifer and Vishny 1993) who seek bribes in return for doing their work and managers, rather than working productively, end up allocating more time and company resources to pay a bribe or kickback (Kaufman et al. 1998; Kaufman and Wei 2000). It has been argued that politicians may be “discriminating against firms with low bargaining power to maximize the private interests of politicians and bureaucrats” (Hellman et al. 2000: p. 2). Thus, stakeholders in the tourism industry are often aware of the inequalities created when firms partake in capture or other forms of corruption since the repercussions on the tourism industry are pervasive.

Corruption is a “hidden crime”. A number of measurement tools attempt to quantify the extent of corruption practices. Hart (2019: p. 8) noted that “some count reported victimization (‘experience’); others survey opinions of experts and broader populations (‘perceptions’) while others track certain types of administrative data”. Two measurement systems selected to be used by the current authors are the Corruption Perception Index (CPI) published by Transparency International (TI) and the International Country Risk Guide (ICRG) published by the PPRS Group.

Transparency International scores countries on how corrupt their public sectors are perceived to be by public officials and politicians. The CPI is a composite index drawing on corruption-related data in expert surveys carried out by a variety of reputable institutions, yielding a CPI score from 0 to 100. Countries that have a high score means they have low perceived corruption such as New Zealand and Denmark with a score of 87 as opposed to Somalia that has a score of 9.

The ICRG has been published since 1988 by the PRS Group and is a “measure of corruption within the political system that threatens foreign investment by distorting the economic and financial environment, reducing the efficiency of government and business by enabling people to assume positions of power through patronage rather than ability, and introducing inherent instability into the political process’’ (Swaleheen 2011: p. 31). This measure looks at political, financial and economic risk variables. Schwindt-Bayer and Tavits (2016) pointed out that the ICRG is a component of CPI and the World Bank’s corruption measurement. The same authors use ICRG as it “offers the most complete time-series for the largest number of countries and it is comparable over time. It is based on expert survey, the content of the measure stays the same for each year, and the scores for one country are independent from those of other countries” (p.46). Furthermore, other researchers (Charron 2011; Yadav 2012), who used ICRG, have maintained that ICRG is comparable across time and countries and can be used in time-series analyses, whereas the CPI provides a snapshot with less capacity to offer year-to-year trends (Campos and Pradhan 2007). Furthermore, in arguing against the use of CPI, Hough (2016) has argued that this measurement system ignores private corruption, like the Libor case in Britain and the VW emissions in the USA.

A limitation of both the CPI and the ICRG is that neither of them is a direct measure of a country’s corruption. Nevertheless, the ICRG is considered “a good proxy of corruption” (Swaleheen 2011: p. 31), whereas the CPI is a composite index of corruption perception. Thus, the current authors, unlike Saha and Yap (2015) and Lv and Xu (2017), have used the ICRG data rather than the CPI.

The negative consequences of corruption have been reported by a number of researchers, and they include hindering economic growth and fostering inefficiency (Shleifer and Vishny 1993; Mauro 1995; Kaufmann and Kraay 2002; Santos et al. 2019). Interestingly, it has been found that corruption increases a firm’s sales and exports in low-income economies but has the opposite effect in middle- and high-income countries (Imran et al. 2019). Furthermore, corruption disrupts international trade and investment as well as public policy (Mauro 1995; Gastanaga et al. 1998; Wei 1999; Zhao et al 2003) and impacts adversely on private sector development, leads to misallocation of resources (Ades and Di Tella 1999) and “hamper innovation efforts” (DiRienzo and Das 2015: p. 54) as it erodes trust and increases transaction costs and, also, undermines mega event support (Santos et al. 2019).

Contrary to the above view, there is an argument that “corruption can actually be very helpful for firms in the tourism industry” (Ekine 2018: p. 48). More specifically, it has been argued that corruption can be beneficial because “greasing the wheel” accelerates processes or sidesteps regulations (Bicchieri and Duffy 1997) or government employees may thus have an incentive to work harder if they ask for bribes (Saha and Yap 2015). Before addressing the nature of the relationship between tourism and corruption, let us first briefly examine a number of studies which have reported both positive and negative correlates of tourism.

### Corruption and tourism relationship

Conflicting views have been expressed regarding the relationship between corruption and tourism. Some (e.g. Huntington 1968) have argued that corruption “greases the wheels” (i.e. it impacts positively on tourism), while others (e.g. Lau and Hazari 2011) have proposed that it “sands the wheels”. Furthermore, other authors have proposed that both the opposite viewpoints are correct and, in fact, the relationship between corruption and tourism is an inverted U-shaped curve (Saha and Yap 2015; Demir and Gozgor 2017; Lv and Xu 2017). Let us examine the three perspectives.

As Harris (2012) reminds us, fraud and corruption are prevalent in the tourism industry with foreign tourists being the main victim. Interestingly, it has also been argued that “greasing the wheels” can increase efficiency (Lien 1986) because it decreases the time waiting in queues (Lui 1985) and can be beneficial in countries “where other aspects of governance are ineffective” (Méon and Sekkat 2005: p. 70). Thus, supporters of this theory maintain that where there is low quality of government in order to reduce the inconvenience caused by unnecessary bureaucracy, greasing of the wheels is necessary. However, it should be noted in this context that corruption is likely to add costs to the price of a travel product since the payment of a bribe will increase the cost of the product and this cost will be passed on the consumer.

Méon and Sekkat (2005: p. 91) have also found that weak law enforcement, an unproductive government and political violence create “sand in the wheels”. For their part, Assaf and Josieassen (2012) argued that where there is corruption, countries are unable to develop their tourism industry despite its cultural and environmental heritage, a view also supported by Yap and Saha (2013).

Support for the argument that an increase in a country’s perceived corruption has a negative effect on its inbound tourism can be found in Poprawe’s (2015) study which established that the effect of corruption on total tourist arrivals is negative in 100 countries. Similarly, Das and Dirienzo (2010) reported that a decrease in corruption affected positively tourism competitiveness in 119 countries. In fact, Lau and Hazari (2011) and Poprawe (2015) did not only find an inverse relationship between corruption and tourism arrivals but quantified the impact on tourism. More specifically, both studies have found that an one-point decrease in corruption, utilizing the CPI, leads to 8% (by Lau and Hazari 2011) and 6–7% (by Poprawe 2015) higher tourist inflows. This relationship proved to be correct in the case of Cyprus in the summer of 2017. Cyprus’ CPI score in January 2017 was higher by 2 points, and utilizing the Cyprus Statistical Service, tourism revenue in 2017 increased by 15.3% compared to the corresponding period in 2016 as tourism arrivals (TA) increased by 14.7% outnumbering the number of tourist arrivals on the island for years; thus, the predictions were proved to be correct. However, the increase in tourism arrivals may be attributable to other politico-socio-economic factors as well at the time, for instance terrorist incidents in neighbouring Egypt and Turkey the year before, diverting a number of tourists to Cyprus. Thus, it can be argued that a country’s corruption score is not the only variable impacting on tourism arrivals.

Focusing on the nonlinear relationship between corruption and tourism demand, Demir and Gozgor (2017) found that the level of *relative corruption* (i.e. the difference between the corruption in the country of the tourist’s departure and the destination country) affects negatively inbound tourism in Turkey; in other words, more tourists visit Turkey from countries which are as corrupt or more corrupt than Turkey. Saha and Yap (2015) utilized data generated with the CPI methodology prior to 2012 (when TI’s CPI score ranged from 0 to 10) and reported that the turning point of most to least corrupt country on the inverted U-curve is 6.7. The inverted U curve illustrates that “corruption level does reduce tourism demand only after a threshold of corruption” (p. 280). Thus, both the “greasing the wheels” and “sanding the wheels” arguments are applicable. Likewise, Lv and Xu (2017) reported a nonlinear relationship between corruption and tourism demand in 62 countries over the period 1998–2011 with the relationship being significant at the 50th and 75th quantiles. Lv and Xu’s study is similar to Saha and Yap as both utilize data which had been created using the old methodology of Transparency International’s Corruption Perception Index. However, in view of the 2012 change of the CPI calculation methodology for calculating a country’s CPI, the applicability of the results reported by Saha and Yap’s (2015) and Lv and Xu (2017) finding concerning the turning point on the inverted U-curve is not applicable post-2011.

## Aims of the present study and methodology

It can be seen from the preceding discussion of the literature that despite efforts by the previous authors to capture the nonlinear relationship between corruption and tourism arrivals, none of them adequately tested the presence of nonlinearity and none have considered the impact of other determinants within the different corruption regimes. For example, what is the effect of inflation on tourism arrivals when corruption is low/high?

The present study aims to fill this gap and contribute to the literature on the relationship between corruption and tourism by analysing the effect of different politico-socio-economic variables on tourism demand in low and high corruption regimes. Drawing also on Hansen (2017) and Kourtellos et al. (2016) where the threshold variable and the regressors are considered endogenous ensuring a statistically adequate model, the aims of the study reported have been to:

1. Ascertain the nature of the relationship between corruption and tourism;
2. Identify any nonlinear effects of corruption on tourism demand and, thus, test the “greasing-the-wheels” and “sanding-the-wheels” hypotheses by finding a threshold point; and finally,
3. Investigate the effect of different politico-socio-economic variables on tourism, within different corruption regimes by means of a Panel Threshold Regression Model.

The current paper utilizes improved methodology to that of other researchers and can be said to contribute significantly to theory since:

1. ICRG data are used rather than the CPI as the former overcomes limitations of CPI.
2. It covers a longer period of time (2001–2018) than other researchers.
3. It utilizes a Panel Threshold Model rather than a linear model with a quadratic specification (Saha and Yap 2015) or a quantile regression (Lv and Xu 2017).
4. The other researchers have not empirically tested adequately for the presence of nonlinearity, and none have considered the impact of other determinants within different corruption regimes.

The documentation of a nonlinear relationship between corruption and tourism demand which can be modelled via a Panel Threshold Regression Model ensures not only a statistically adequate model but also confirms the theoretical predictions of Mauro (1995, 2002) for the presence of multiple equilibria in corruption and economic growth and similarly between corruption and tourism, since tourism constitutes a share of GDP.

### Data description and the tourism demand model

Regarding the variables used for the statistical analysis, the authors utilized data from the World Tourism Organization (WTO) and particularly Tourist Arrivals and Tourism Receipts as proxies for the tourism demand. For the tourism demand determinants, data are used from (a) World Bank—World Development Indicators (WDI) and more precisely macroeconomic variables, GDP per capita, Trade Openness and Inflation, and (b) the International Country Risk Guide (ICRG) and specifically, measures of political (corruption, law and order, civil disorder, civil war, ethnic tensions, foreign pressures, military in politics, and religious tensions) and economic institutions (economic and financial risk rating and the risk for exchange rate stability). A detailed description of the variables and their source is given in Table 4 in the Appendix, whereas Table 5 presents the summary statistics for the pooled data.

GDP per capita measures the propensity and economic performance of a country and has been extensively used in studies examining tourists’ arrivals (Leitão 2010; Poprawe 2015; Santana-Gallego et al. 2016). Surugiu et al. (2011) found a positive relationship between tourism demand and GDP of tourists’ origin country, indicating the impact of GDP on tourists’ decision for travelling. Openness to trade is a variable that examines how a country’s trade connectedness to international markets influences tourists’ arrivals to a destination. In support of Ibrahim (2011) writing about Egypt, Poprawe (2015) also reported that openness to trade impacts positively on tourism arrivals. Similarly, Leitão (2010) found that bilateral trade influenced significantly tourism arrivals in Portugal. However, openness to trade was found to be an insignificant variable with positive impact on tourism arrivals in Malaysia (Habibi et al., 2009). Unfortunately, however, on the basis of the available information provided in the studies it is not possible to account for the conflicting findings concerning the effect of openness to trade in different countries.

Predictably, perhaps, Meo et al. (2018) found in their study that an increase in inflation affects tourism demand negatively as tourists find a destination too expensive for them, while Naidu et al. (2017) reported that inflation affects negatively the tourism output in the medium and long term. Also as might be expected, exchange rate risk has been found by a number of studies to impact adversely on tourism arrivals (Garin-Munoz and Amaral 2000; Salman 2003; Hanafiah and Harun 2010). Examining the impact of the exchange rate between Mauritian price and US dollar using relative prices, Khadaroo and Seetanah (2007) found that relative price influences the decision of tourists travelling from Asia and Africa but not for tourists from Europe and America, emphasizing the importance of maintaining a relatively stable exchange rate in order to attract tourist arrivals (De Vita 2014).

The way political institutions in a country function undoubtedly impacts on the international image of that country. Neumayer (2004) argued that tourists are willing to travel to destinations where they will feel safe to enjoy their holidays without fear of bodily harm. Similarly, Feridun (2011) and Altindag (2014) support the existence of an inverse relationship between violent crimes and incoming tourists. Henderson (2003) and Yap and Saha (2013) reported that military involvement in politics can be a disincentive for tourists as it can result in lack of free movement in the nation and civil rights. However, a number of other authors (Larsen et al. 2009; Boakye 2010; Papathanassis 2016) have maintained that tourists are likely to return to holiday destinations and recommend them to others regardless of crime and other safety concerns to potential tourists. In support of that point of view, when Nonthapot and Lean (2013) examined the political crisis of Lao over the period 2008–2010, they found no effect on tourism arrivals.

Following Durlauf et al. (2008) and other scholars (Abed and Gupta 2003; Chong and Gradstein 2007; Yadav and Mukherjee 2016), the authors have rescaled corruption (and all ICRG variables), between 0 and 1 (0 ≤ x ≤ 1), where the lowest point 0 indicates low corruption and the highest point 1, high corruption, “for comparability and ease of interpretation” (Chan et al. 2019).

Finally, countries that did not satisfy the selection criteria (in terms of the presence of the variables addressed and time span considering that the estimation requires a balanced panel) were excluded, leaving 83 countries for the period 2001–2018 (see Table 6 in the Appendix).

Following the literature (Song and Li 2008; Poprawe 2015; Saha and Yap 2015; Lv and Xu 2017), Tourist Arrivals (TA) was set as the baseline for the Tourism Demand Model which is defined as the number of tourists who travel to a country other than that in which they have their usual residence, which is outside their usual environment, for no more than 12 months and whose main purpose in visiting is other than an activity paid for within the country visited. Then, using a standard Fixed Effects balanced panel over the period 2001–2018 and for 83 countries the suggested Linear Tourism Demand Model will take the form of

1 $$ y_{it} = \mu_{i} + \beta ^{\prime}x_{it} + e_{it} $$

where the dependent variable $y_{it}$ is a scalar and measures the annual growth rate of tourist arrivals, $x_{it}$ is a $k \times 1$ vector of tourism demand determinants, $\beta$ is a $k \times 1$ vector of unknown parameters, $e_{it}$ is an *i.i.d.* error term for country *i* = 1*,* 2*,..., N* and time *t* = 1*,* 2*,...,T.*

To address any issues of non-stationarity, we follow Hadri (2000), Levin et al. (2002) and Im et al. (2003) and implement three alternative panel unit root tests. According to the results, all the variables in (1), which are calculated in annual growth rates, are stationary.^1^

In the spirit of Hansen (1999, 2000, 2017), Caner and Hansen (2004) and Kourtellos et al. (2016), we apply the static threshold GMM of Seo and Shin (2016), and the Panel Fixed Effects Threshold Tourism Demand Model generalizes the linear model in (1) by allowing for the presence of multiple regimes. In particular,

2 $$ y_{it} = \left\{ {\begin{array}{*{20}c} {\mu_{i} + \beta_{1}^{^{\prime}} x_{it} + e_{it} , \quad q_{it} \le \gamma } \\ {\mu_{i} + \beta_{2}^{^{\prime}} x_{it} + e_{it} ,\quad q_{it} > \gamma } \\ \end{array} } \right. $$

where $\gamma$ is the scalar threshold parameter or sample split value and ($\beta_{1}^{^{\prime}} , \beta_{2}^{^{\prime}} )$ is the vector of regression coefficients for the low and high regime, respectively. Therefore, the threshold model sorts the data into two groups of observations based on whether the threshold variable $q_{it}$, namely corruption, is above or below the threshold parameter (sample split) $\gamma .$ Alternatively, (2) can be expressed in a single equation as

3 $$ y_{it} = \mu_{i} + \beta_{1}^{^{\prime}} x_{it} {\rm I}\left( {q_{it} \le \gamma } \right) + \beta_{2}^{^{\prime}} x_{it} {\rm I}\left( {q_{it} > \gamma } \right) + e_{it} $$

where $I\left( . \right)$ is the indicator function. In this setting, we allow for the threshold variable $q_{it}$ and the set of the determinants $x_{it}$ to be endogenous and appropriately instrumented using their lag values.

Estimation of the Threshold Tourism Demand Model requires first to determine if the threshold effect is statistically significant. Seo and Shin (2016) propose a bootstrap test for the null hypothesis of a linear model based on a supremum Wald statistic.

In practice, the presence of a nonlinear relationship between corruption and tourism demand is first tested by estimating the particular threshold/turning point, and then, uncovering the effect of corruption and all the other variables on tourism demand under the different corruption regimes by estimating a Panel Threshold Model.

## Empirical results

The first aim of the study is to identify the nature of the relationship, linear or nonlinear, between corruption and tourism demand using a threshold test.

Table 1 shows the results of the threshold test for tourism arrivals considering the presence of one and two thresholds. The first column of the table shows the number of thresholds/splits under consideration for corruption, then the corresponding p value for the null hypothesis of a linear model against the alternative of a threshold, the threshold estimate and the sample sizes of the two regimes.

**Table 1:** Threshold tests and threshold estimates—tourist arrivals

Threshold variable	*P* value	Threshold	n1	n2
Corruption (single threshold)	0.0014	0.5208	634	860
Corruption (double threshold)	0.6583	0.5903	–	–

Table 1 presents the threshold test with the corresponding *p* value and threshold estimate for tourist arrivals for the null hypothesis of a linear model against the alternative of a threshold.

According to the results (*p* value = 0.0014), the linear model null hypothesis is strongly rejected for the presence of one threshold/split. The authors also report the estimated corruption threshold point which is 0.5208, as well as, the number of observations included in the low (below 0.5208) and the high corruption regime (above 0.5208) which are 634 and 860, respectively. On the other hand, the p value for the presence of a second threshold/split equals to 0.6583, a result which was robust under different model specifications. Justifiably, then, it can be inferred that “there is a nonlinear relationship between corruption and tourism arrivals” confirming Saha and Yap (2015: p. 276) based on only one threshold in all of the regression relationships.

Table 2 shows the threshold regression estimation for the two regimes. The second column in Table 2 illustrates the regression coefficient and the corresponding robust standard errors for the Linear Fixed Effects Panel Model (which was decisively rejected) for comparison reasons, whereas the remaining columns present the regression coefficients and robust standard errors for the low and high corruption regime, respectively, in the context of a Panel Threshold Model. Regarding the effect of corruption and all the other politico-socio-economic variables on tourism arrivals in the linear Fixed Effects model, corruption has a strong negative effect on tourism arrivals (− 0.2532) with significance of 5% which supports the sand-in-the-wheel hypothesis, i.e. that high levels of corruption result in lower tourists’ arrivals in destinations. The results in Table 2 also provide support for the hypotheses that tourists are significantly less likely to visit a country with civil war, ethnic and religious tensions, increased financial risk and inflation. However, tourists are more likely to visit a destination with higher GDP per capita or openness to trade.

**Table 2:** Threshold regression—tourist arrivals

Variable	Linear coef	Low corruption Regime $\le$0.5208 coef	High corruption Regime $>$0.5208 Coef	Low corruption Regime $\le$0.5208 coef	High corruption Regime $>$0.5208 coef	Low corruption Regime $\le$0.5208 coef	High corruption Regime $>$0.5208 coef
Corruption	− 0.2532** (0.1266)	0.3841*** (0.1431)	− 0.3267*** (0.1155)	0.4034*** (0.1541)	− 0.3996*** (0.1262)	0.4393*** (0.1347)	− 0.3677*** (0.1240)
Civil disorder	0.0619 (0.0803)	−	−	− 0.1267 (0.1287)	− 0.0591 (0.1235)	0.1422 (0.0937)	0.0789 (0.1017)
Civil war	− 0.5712*** (0.1303)	−	−	− 0.2043 (0.2121)	− 0.8083*** (0.1390)	− 0.1934 (0.1565)	− 0.9038*** (0.1136)
Economic risk rating	− 0.0442 (0.1339)	−	−	− 0.3093 (0.1989)	− 0.8249*** (0.2442)	− 0.2720 (0.2426)	− 0.9272*** (0.1962)
Ethnic tensions	− 0.9625*** (0.2461)	−	−	− 0.8487*** (0.2341)	− 0.9790*** (0.2212)	− 0.7895*** (0.1771)	− 0.9825*** (0.1796)
Financial risk rating	− 0.2970* (0.1709)	−	−	− 0.3098** (0.1301)	− 0.7701*** (0.2115)	− 0.4425** (0.1737)	− 0.5547*** (0.1787)
Foreign pressures	0.0795 (0.1108)	−	−	− 0.3212 (0.2298)	− 0.6549*** (0.1345)	− 0.2056 (0.2871)	− 0.7804*** (0.1123)
Law and order	0.3039 (0.2623)	−	−	− 0.2353 (0.2922)	− 0.4980* (0.2630)	0.1279 (0.2209)	− 0.5154** (0.2118)
Military in politics	− 0.2698 (0.1709)	−	−	− 0.1521 (0.2220)	− 0.6038*** (0.1882)	0.1996 (0.1689)	− 0.4217*** (0.1549)
Religious tensions	− 0.8268*** (0.2658)	−	−	− 0.4983*** (0.1903)	− 0.6787*** (0.2216)	− 0.5892*** (0.1446)	− 0.9089*** (0.1787)
Risk for exchange rate stability	− 0.0149 (0.0770)	−	−	− 0.2366 (0.1508)	− 0.3441*** (0.1095)	0.0200 (0.0902)	− 0.2988*** (0.0967)
GDP PC	1.6509*** (0.0829)	1.2340*** (0.0675)	0.9537*** (0.0956)	−	−	1.3067*** (0.0980)	1.2283*** (0.0809)
Open	0.2546*** (0.0564)	0.2479*** (0.0434)	0.1289 (0.0906)	−	−	0.1846*** (0.0458)	0.1255* (0.0725)
Inflation (%)	− 0.0053*** (0.0015)	− 0.0084 (0.0347)	− 0.0031*** (0.0012)	−	−	− 0.0057 (0.0048)	− 0.0021* (0.0012)

Table presents the linear and the threshold regression model estimation results for tourist arrivals. The second column includes the coefficient and robust standard errors (in parentheses) for the linear Panel Fixed Effects model. The remaining columns present the threshold regression estimation results (coefficients and robust standard errors) for different model specifications. In all model estimations, the variables are considered endogenous (including the threshold variable) and instrumented using their lag-values. All specifications always include an intercept and a time trend

Asterisks denote statistical significance at the 1% (***), 5% (**) and 10% (*) level

Applying the Panel Threshold Model emphasizes the presence of the parameter heterogeneity in the sense that the effect of corruption on tourism arrivals is not exactly negative as in the linear model, but, rather, it depends on the level of corruption (below or above 0.5208), yielding a nonlinear relationship between corruption and tourism arrivals in the form of an inverted-U-shape curve.

Considering the model specification with all the explanatory variables included, all else being equal, higher levels of corruption result in lower tourism arrivals ( − 0.3677) for countries in the high corruption regime (above 0.5208), as the sand-in-the-wheel view suggests. However, for countries with better quality institutions, particularly countries in the low corruption regime (below 0.5208), corruption has a significant positive effect (0.4393) on tourism arrivals emphasizing the grease-in-the wheel hypothesis, thus achieving the second aim of the paper. Importantly and in support of Saha and Yap (2015: p. 280), the results indicate the threshold point where corruption stops being the grease “in the wheel” and becomes the sand “in the wheel” with adverse effect on tourism arrivals. It is also important to note that the negative coefficient of corruption in the high corruption regime is notably larger ( − 0.3677) than the corruption coefficient in the linear model ( − 0.2532), indicating the detrimental effects of bad institutions on tourism demand. This is illustrated in Table 2 where a calculation of the average arrivals in the low corruption regime is twice those in the high corruption regime, i.e. average tourism arrivals for low corruption regimes is 12,854,000 and 6,646,000 for high corruption regimes.

The third aim of the study is to investigate the effect of various politico-socio-economic variables on tourism, within different corruption regimes in the context of a Panel Threshold Regression Model. The results in Table 2 support the presence of parameter heterogeneity not only of corruption on tourism demand, but also of the other determinants as well.

Tourists are less likely to visit countries characterized by internal conflicts, economic and political risks. In particular, based on the results presented in columns 7 and 8 in Table 2, tourists will avoid countries with civil wars, increased economic, exchange rate and law and order risks, with military involvement in politics and foreign pressures, all statistically significant in the high corruption regime. Further, ethnic and religious tension, as well as increased financial risk, reduce tourist arrivals significantly, especially in the high corruption regime. Focusing on the effect of the macroeconomic variables, tourists are more likely to visit a country with higher GDP per capita and more open to trade, a result which is larger and stronger in the low corruption regime. Finally, inflation has a negative effect on tourism demand but only the high corruption regime.

Thus, the paper has achieved its third aim as well, in identifying the politico-socio-economic factors impacting tourism in different corruption regimes (see Table 3). This is a significant contribution to theory as no other researchers have found this relationship within the two corruption regimes.

**Table 3:** Countries within the corruption regimes

Country	Low corruption regime	High corruption regime
Angola		2001–2018
Armenia		2001–2018
Austria	2001–2018	
Bahamas	2001–2018	
Belarus		2001–2018
Belgium	2001–2018	
Bolivia	2001	2002–2018
Brazil	2003, 2009–2011	2001–2002, 2004–2008, 2012–2018
Bulgaria	2017–2018	2001–2016
Burkina Faso		2001–2018
Canada	2001–2018	
Chile	2001–2002, 2005–2018	2003–2004
China		2001–2018
Colombia	2003–2005, 2009–2011	2001–2002, 2006–2008, 2012–2018
Congo (Republic)	2001–2002	2003–2018
Costa Rica	2001–2002, 2015–2017	2003–2014, 2018
Croatia	2001–2004, 2010–2011, 2015–2018	2005–2009, 2012–2014
Cyprus	2001–2018	
Denmark	2001–2018	
Dominican Republic	2001	2002–2018
Egypt		2001–2018
Estonia	2001–2018	
Finland	2001–2018	
France	2001–2018	
Gambia	2001–2004	2005–2018
Germany	2001–2018	
Greece	2001	2002–2018
Guyana	2001–2005	2006–2018
Haiti		2001–2018
Hong Kong	2001–2018	
Hungary	2001–2018	
Iceland	2001–2018	
India		2001–2018
Ireland	2002–2003, 2005–2018	2001, 2004
Israel	2001–2018	
Italy	2001, 2018	2002–2017
Jordan	2001–2011, 2015–2018	2012–2014
Latvia	2015–2017	2001–2014, 2018
Lithuania	2001, 2015–2018	2002–2014
Luxembourg	2001–2018	
Madagascar	2001–2011	2012–2018
Malawi	2001	2002–2018
Malaysia		2001–2018
Mali	2003	2001–2002, 2004–2018
Malta	2001–2018	
Mexico	2001	2002–2018
Moldova		2001–2018
Mongolia		2001–2018
Morocco	2001–2004, 2006–2011, 2017–2018	2005, 2012–2016
Mozambique	2001–2018	
Myanmar		2001–2018
Netherlands	2001–2018	
New Zealand	2001–2018	
Nicaragua	2001	2002–2018
Niger		2001–2018
Norway	2001–2018	
Oman	2015–2018	2001–2014
Panama		2001–2018
Paraguay		2001–2018
Peru	2001	2002–2018
Philippines		2001–2018
Poland	2013–2018	2001–2012
Portugal	2001–2018	
Qatar	2013–2018	2001–2012
Saudi Arabia	2015–2018	2001–2014
Sierra Leone	2001	2002–2018
Singapore	2001–2018	
Slovenia	2001–2018	
South Africa	2001, 2010–2011	2002–2009, 2012–2018
Spain	2001–2018	
Sri Lanka	2001–2004	2005–2018
Sudan		2001–2018
Tanzania	2007–2009	2001–2006, 2010–2018
Thailand		2001–2018
Togo		2001–2018
Trinidad and Tobago	2001	2002–2018
Tunisia	2001	2002–2018
Turkey		2001–2018
Ukraine		2001–2018
United Kingdom	2001–2018	
United States	2001–2018	
Uruguay	2001–2018	
Zambia	2005–2011	2001–2004, 2012–2018

## Robustness

In testing the robustness of the results obtained, the authors have carried out multiple robustness tests reported in the Online Appendix.

In the first, the authors compared the corruption regime classification (low—below 0.5208, or high—above 0.5208) of each country (see Table 3) with Transparency International’s CPI ranking for the year 2018, which is the last year in the data set tested for this paper. It was interesting to find that countries ranked by Transparency International in their 2018 CPI with a score higher than 45 were also found by the current authors to be ranked as countries in the low corruption regime. Conversely, countries like Armenia, Algeria, Tanzania, Iran, Russia and Sudan that had a score below 45 were ranked in the high corruption regime by the authors.

To further test for robustness, tourist arrivals was replaced with tourism receipts which include all transactions related to the consumption of goods and services by international visitors. Based on the findings from Tables 1 and 2 in the Online Appendix, similar results were obtained, with the difference in the threshold being 0.5000 rather than 0.5208 as eight countries were missing tourism receipts data and, consequently, the sample is only 75 countries in the robustness test rather than 83.

To establish further our results, the third robustness exercise includes replacing corruption from ICRG with the Corruption Control Index from the Worldwide Governance Indicators. The control of corruption index captures perceptions of the extent to which public power is exercised for private gain, including both petty and grand forms of corruption, as well as “capture” of the state by elites and private interests. It also measures the strength and effectiveness of a country’s policy and institutional framework to prevent and combat corruption. The variable ranges fro  − 2.5 indicating most corrupt/least effective to 2.5 indicating least corrupt/most effective.

According to the results in Table 3 in the Online Appendix, the linear model null hypothesis is strongly rejected for the presence of one threshold/split (*p* value 0.0033), whereas the presence of a second threshold is rejected (*p* value 0.4513). The estimated corruption threshold point is 0.2800 with 909 observations in the high corruption regime and 585 in the low corruption regime.

Table 4 in the Online Appendix reports the estimated threshold regression model for tourist arrivals and corruption control index. The results confirm the previous findings regarding the positive effect of corruption in the low corruption regime and the corresponding negative impact on tourist arrivals in the high corruption regime.

As a last robustness exercise, we address the issue of theory uncertainty by implementing Bayesian model averaging (BMA) since the effect of a particular regressor including corruption may vary across different model specifications. BMA was developed and studied from different scholars including Leamer (1978), Draper (1995), Kass and Raftery (1995), Raftery et al. (1997), Brock and Durlauf (2001), among others. Model averaging constructs estimates using information from all candidate models, and in particular, it forms a weighted average of model specific estimates where the weights are given by the posterior model probabilities. Based on Eq. (1), the BMA estimator takes the form of a weighted average of model-specific coefficient estimates:

4 $$ \hat{\beta }_{{{\rm B}{\rm M}{\rm A}}} = \mathop \sum \limits_{m = 1}^{M} w_{m} \hat{\beta }_{m} $$

where $M = \left\{ {M_{1} , \ldots ,M_{M} } \right\}$ denotes the model space and the weights $W = \left\{ {w_{1} , \ldots ,w_{M} } \right\}$ reflect the evidentiary support for each model given the data. The weights W are given by the posterior model probabilities computed using the Bayes’ rule, such that each weight is the product of the integrated likelihood of the data given a model and the prior probability for a model. In our estimation, we assume a uniform model prior such that the prior probability that any variable is included in the true model is taken to be 0.5. The corresponding model averaging variance estimator is given by

5 $$ \hat{V}_{{{\rm B}{\rm M}{\rm A}}} = \mathop \sum \limits_{m = 1}^{M} w_{m} \hat{V}_{m}^{\beta } + \mathop \sum \limits_{m = 1}^{M} w_{m} \left( {\hat{\beta }_{m} - \hat{\beta }_{{{\rm B}{\rm M}{\rm A}}} } \right)^{2} $$

Using the posterior mean and variance $\hat{\beta }_{{{\rm B}{\rm M}{\rm A}}}$ and $\hat{V}_{{{\rm B}{\rm M}{\rm A}}}$, we calculate posterior t-statistics and interpret them in the classical sense. In addition, we also report the posterior probability of inclusion (PIP) for each regressor which is computed as the sum of posterior probabilities of the models which contain that variable. Following Kass and Raftery (1995), we interpret the values of PIP as follows: PIP < 50% indicates no evidence for an effect, 50% < PIP < 75% indicates weak evidence for an effect, 75% < PIP < 95% indicates positive evidence for an effect, 95% < PIP < 99% indicates strong evidence for an effect, and 99% < PIP < 100% indicates decisive evidence for an effect. Table 5 in the Online Appendix presents BMA results for the linear and threshold model for tourist arrivals, which confirm the previous findings. Notably, corruption is still positive and statistically significant in the low corruption regime and negative and also significant in the high corruption regime with posterior probability of inclusion to be one.

## Conclusions

Tourism has increased significantly over the years, and many countries today rely on it to drive their economic growth, create jobs and generate tax revenue. Studies have examined the impact on tourism arrivals of different factors, including corruption. Given that many countries are plagued by corruption, and at the same time they rely on tourism for their economic growth, knowing the exact nature of the relationship between corruption and tourism becomes imperative.

The findings indicate that corruption influences tourism demand both directly and indirectly. As confirmed by the present study, the effect of corruption on tourism arrivals can be either positive or negative, suggesting that the direct relationship between these variables is nonlinear, an issue that has received inadequate attention in the tourism demand literature. In support of Saha and Yap (2015: p. 276) and by (a) adopting the Panel Threshold Model and (b) using data for 83 countries for the period between 2001 and 2018, the data analysis yielded a nonlinear relationship between corruption and tourism arrivals. The second aim of the paper was to identify the threshold point where corruption stops greasing the wheel and becoming sanding the wheel, thus decreasing tourism arrivals.

In addition to determining the nature of the relationship between corruption and tourism demand, the third aim of the paper was to investigate the effect of various politico-socio-economic variables in the different corruption regimes and how they each affect tourism arrivals. Low corruption regimes are characterized by effective mechanisms for promoting integrity and preventing corruption by means of good anti-corruption legislation, good administration, integrity of civil servants and civil society participation.

Thus, tourists are significantly more likely to visit a low corruption regime country where the positive effect of GDP and openness to trade is stronger than the high corruption regime. By contrast, tourist arrivals are affected negatively from civil war, economic risk rating, foreign pressures, law and order, military in politics, exchange rate risk and inflation, factors which are significant only in countries with high corruption.

It can, thus, be justifiably argued that the study reported has achieved all its stated aims: it rejected the null hypothesis of no threshold (linear model) relationship between tourism and corruption, confirming also the theoretical predictions of Mauro for the presence of multiple equilibria in corruption and economic growth and similarly between corruption and tourism; estimated the turning point of corruption regime countries; and, unlike previous studies, went on to examine not only the effect of corruption on tourism arrivals, but also the effect of various different politico-economic-socio variables under both low and high corruption regimes.

Furthermore, it can also be rightly argued that the study reported fills a research gap in a number of ways. Firstly, concerning methodology, it improves on the existing empirical studies (Saha and Yap 2015; Demir and Gozgor 2017 and Lv and Xu 2017), by applying a threshold analysis (see Caner and Hansen 2004; Hansen 2017, 2000, 1999 and Kourtellos et al. 2016) and particularly the static threshold GMM of Seo and Shin (2016), to a large panel of countries for the first time. The present study shows statistically that the null hypothesis of a linear model is decisively rejected and the nonlinear relationship between corruption and tourism arrivals has been demonstrated using a Panel Threshold Model with one threshold point. Thus, the study provides new insights into the relationship between corruption and other variables. A key characteristic of this method is that the “variable of interest (corruption) is observable, but the position of the threshold is not known” (Polemis and Stengos 2018: p. 99) and it is based on a sample-splitting framework.

An important critique of previous studies was the subjective pre-selection of threshold value. The threshold analysis used in the current paper is not subject to that criticism as it follows an objective strategy for identifying and testing changes in the slope. In addition, this methodology allows testing for additional sample splits (thresholds), thus also exploring the presence of more than two corruption regimes (multiple corruption regimes).

A critical appraisal of the existing literature and the findings obtained in the present study point to a number of policy implications. Firstly, legislatures, technocrats and entities like Chambers of Commerce, tourism industry regulators or those responsible for formulating national strategies for economic growth ought to first tackle corruption if they wish to increase tourism arrivals. Secondly, addressing corruption effectively means implementing a holistic approach meaningfully by involving the stakeholders (Krambia-Kapardis 2016; Azim and Kluvers 2019) and including adherence to a code of conduct, ethical policies, implementation of social responsibility policies, applying ethical and transparent lobbying and implementing whistle-blowing procedures. Support for such an approach has also been voiced by Lyrio and Lunken (2018) who encourage tourists to come forward more when they witness corrupt practices on their holidays. By addressing corruption and endeavouring to place a country in the low corruption regime, tourism arrivals would be expected to increase when the other politico-socio-economic variables are taken into account.

### Electronic supplementary material

Below is the link to the electronic supplementary material.

Supplementary file1 (DOCX 29 kb)

## Data Availability

Data are available upon request.

## Appendix

See Tables

**Table 4:** Variable description and data sources

Variable	Description
Tourist arrivals	International inbound tourists (overnight visitors) are the number of tourists who travel to a country other than that in which they have their usual residence, but outside their usual environment, for a period not exceeding 12 months and whose main purpose in visiting is other than an activity remunerated from within the country visited. When data on number of tourists are not available, the number of visitors, which includes tourists, same-day visitors, cruise passengers, and crew members, is shown instead. Sources and collection methods for arrivals differ across countries. In some cases, data are from border statistics (police, immigration and the like) and supplemented by border surveys. In other cases, data are from tourism accommodation establishments. For some countries, the number of arrivals is limited to arrivals by air and for others to arrivals staying in hotels. Some countries include arrivals of nationals residing abroad, while others do not. Caution should thus be used in comparing arrivals across countries. The data on inbound tourists refer to the number of arrivals, not to the number of people traveling. Thus, a person who makes several trips to a country during a given period is counted each time as a new arrival. In a logarithmic form. *Source: World Tourism Organization (WTO). *http://www2.unwto.org/en*. Data access is restricted to subscribers*
Tourism Receipts	For destination countries, receipts from international tourism count as exports in the balance of payments (travel) of each country and cover all transactions related to the consumption of goods and services by international visitors, such as accommodation, food and drink, fuel, domestic transport, entertainment and shopping. They include transactions generated by same-day as well as overnight visitors. Receipts from same-day visitors can be substantial, especially in the case of neighbouring countries where shopping accounts for a large amount of spending by cross-border, same-day visitors. International tourism receipts (travel) do not include receipts from international passenger transport contracted from companies outside the travellers’ countries of residence. *Source: World Tourism Organization (WTO). *http://www2.unwto.org/en*. Data access is restricted to subscribers*
Corruption	A measure of corruption within the political system that is a threat to foreign investment by distorting the economic and financial environment, reducing the efficiency of government and business by enabling people to assume positions of power through patronage rather than ability and introducing inherent instability into the political process. Between 0 (high corruption) and 6 (low corruption). Rescaled between 0 (very clean) and 1 (highly corrupt).*Source: International Country Risk Guide (ICRG). *https://epub.prsgroup.com/products/icrg-historical-data*. Data access is restricted to subscribers*
Control of Corruption Index	Control of corruption captures perceptions of the extent to which public power is exercised for private gain, including both petty and grand forms of corruption, as well as “capture” of the state by elites and private interests. It also measures the strength and effectiveness of a country’s policy and institutional framework to prevent and combat corruption. Ranges from -2.5 (most corrupt/least effective) to 2.5 (least corrupt/most effective) Source: *Worldwide Governance Indicators. *https://info.worldbank.org/governance/wgi*. Data access is open*
Civil Disorder	“The potential risk to governance or investment from mass protest, such as anti-government demonstrations, strikes, etc. Between 0 (high risk) and 4 (low risk)”. Rescaled between 0 (low risk) and 1 (high risk). *Source: International Country Risk Guide (ICRG)* https://epub.prsgroup.com/products/icrg-historical-data*. Data access is restricted to subscribers*
Civil War	The actual or potential risk of civil war (where a rebel force, which holds territory, is in armed conflict with the security forces of the government, and where both forces are citizens of the state in which the conflict occurs). Between 0 (high risk) and 4 (low risk). Rescaled between 0 (low risk) and 1 (high risk). *Source: International Country Risk Guide (ICRG). *https://epub.prsgroup.com/products/icrg-historical-data*. Data access is restricted to subscribers*
Economic Risk Rating	A means of assessing a country’s current economic strengths and weaknesses. In general, where strengths outweigh weaknesses, a country will show low risk, and where weaknesses outweigh strengths, the economic risk will be high. To ensure comparability between countries, risk components are based on accepted ratios between the measured data within the national economic/financial structure, and then the ratios are compared, not the data. Risk points are assessed for each of the component factors of GDP per head of population, real annual GDP growth, annual inflation rate, budget balance as a percentage of GDP, and current account balance as a percentage of GDP. Risk ratings range from a high of 50 (least risk) to a low of 0 (highest risk), though lowest de facto ratings are generally near 15. Rescaled between 0 (low risk) and 1 (high risk). *Source: International Country Risk Guide (ICRG). *https://epub.prsgroup.com/products/icrg-historical-data*. Data access is restricted to subscribers*
Ethnic Tensions	A measure of the degree of tension attributable to racial, national, or language divisions. Between 0 and 6. Lower ratings near 0 (higher risk) are given to countries where tensions are high because opposing groups are intolerant and unwilling to compromise. Higher ratings, near 6, are given to countries where tensions are minimal, even though such differences may still exist. Rescaled between 0 (low risk) and 1 (high risk). *Source: International Country Risk Guide (ICRG). *https://epub.prsgroup.com/products/icrg-historical-data*. Data access is restricted to subscribers*
Financial Risk Rating	A means of assessing a country’s ability to pay its way by financing its official, commercial and trade debt obligations. To ensure comparability between countries, risk components are based on accepted ratios between the measured data within the national economic/financial structure, and then the ratios are compared, not the data. Risk points are assessed for each of the component factors of foreign debt as a percentage of GDP, foreign debt service as a percentage of exports of goods and services (XGS), current account as a percentage of XGS, net liquidity as months of import cover, and exchange rate stability. Risk ratings range from a high of 50 (least risk) to a low of 0 (highest risk), though lowest de facto ratings are generally near 20. Rescaled between 0 (low risk) and 1 (high risk). *Source: International Country Risk Guide (ICRG). *https://epub.prsgroup.com/products/icrg-historical-data*. Data access is restricted to subscribers*
Foreign Pressures	Actual or potential risk posed by pressures brought to bear on the government by one or more foreign states to force a change of policy. Such pressures can range from diplomatic pressures, through suspension of aid and/or credits, to outright sanctions. Between 0 (high risk) and 4 (low risk). Rescaled between 0 (low risk) and 1 (high risk). Source: International Country Risk Guide (ICRG). https://epub.prsgroup.com/products/icrg-historical-data*. Data access is restricted to subscribers*
Law and Order	Two measures comprising one risk component. Each sub-component equals half of the total. The “law” sub-component assesses the strength and impartiality of the legal system, and the “order” sub-component assesses popular observance of the law. Between 0 (high risk) and 6 (low risk). Rescaled between 0 (low risk) and 1 (high risk). *Source: International Country Risk Guide (ICRG). *https://epub.prsgroup.com/products/icrg-historical-data*. Data access is restricted to subscribers*
Military in Politics	A measure of the military’s involvement in politics. Since the military is not elected, involvement, even at a peripheral level, diminishes democratic accountability. Military involvement might stem from an external or internal threat, be symptomatic of underlying difficulties, or be a full-scale military takeover. Over the long term, a system of military government will almost certainly diminish effective governmental functioning, become corrupt, and create an uneasy environment for foreign businesses. Between 0 and 6. Overall, lower risk ratings (0) indicate a greater degree of military participation in politics. Rescaled between 0 (low participation) and 1 (high participation). *Source: International Country Risk Guide (ICRG). *https://epub.prsgroup.com/products/icrg-historical-data*. Data access is restricted to subscribers*
Religious Tensions	A measure of religious tensions arising from the domination of society and/or governance by a single religious group—or a desire to dominate—in a way that replaces civil law by religious law, excludes other religions from the political/social processes, and suppresses religious freedom or expressions of religious identity. The risks involved range from inexperienced people imposing inappropriate policies to civil dissent or civil war. Between 0 (high tensions) and 6 (low tensions). Rescaled between 0 (low tensions) and 1 (high tensions). *Source: International Country Risk Guide (ICRG). *https://epub.prsgroup.com/products/icrg-historical-data*. Data access is restricted to subscribers*
Risk for Exchange Rate Stability	Ranging from high % change of either 0.0—9.9 appreciation or depreciation of 0.1–4.9 with risk points at 10.0, to a midpoint of either appreciation at 50.0 + or depreciation of 30.0—34.9 with risk points at 5.0 to a low depreciation of 100.0 + with 0.0 points. Between 0 and 10. The higher the points (at 10), the lower the risk for appreciation/depreciation. Rescaled between 0 (low risk) and 1 (high risk). *Source: International Country Risk Guide (ICRG). *https://epub.prsgroup.com/products/icrg-historical-data*. Data access is restricted to subscribers*
GDPpc	GDP per capita is gross domestic product divided by midyear population. GDP is the sum of gross value added by all resident producers in the economy plus any product taxes and minus any subsidies not included in the value of the products. It is calculated without making deductions for depreciation of fabricated assets or for depletion and degradation of natural resources. Data are in constant 2010 U.S. dollars. In a logarithmic form. *Source: World Bank, World Development Indicators (WDI).* http://datatopics.worldbank.org/world-development-indicators/. *Data access is open*
Open	Trade is the sum of exports and imports of goods and services measured as a share of gross domestic product *Source: World Bank, World Development Indicators (WDI). *http://datatopics.worldbank.org/world-development-indicators/*.** Data access is open*
Inflation (%)	Inflation as measured by the consumer price index reflects the annual percentage change in the cost to the average consumer of acquiring a basket of goods and services that may be fixed or changed at specified intervals, such as yearly. The Laspeyres formula is generally used. *Source: World Bank, World Development Indicators (WDI). *http://datatopics.worldbank.org/world-development-indicators/*. Data access is open*

4,

**Table 5:** Descriptive statistics

Variable	Mean	Standard diviation	Min	Max
Tourist arrivals	9280.13	15,430.8	16.000	89,322.0
Tourist arrivals (log)	7.8293	1.8399	2.7726	11.4000
Tourism receipts	9102.20	19,144.3	3.0000	214,680.0
Tourism receipts (log)	7.7674	1.8622	1.0986	12.2769
Corruption	0.5183	0.2043	0.0000	0.9167
Control of corruption index	0.1963	1.0467	− 1.7200	2.4700
Civil disorder	0.3126	0.1428	0.0000	0.8750
Civil war	0.0650	0.1345	0.0000	1.0000
Economic risk rating	0.2783	0.1036	0.0000	0.6025
Ethnic tensions	0.3155	0.1985	0.0000	0.9167
Financial risk rating	0.2565	0.1007	0.0308	0.6425
Foreign pressures	0.2632	0.1559	0.0000	1.0000
Law and order	0.3384	0.2116	0.0000	0.8333
Military in politics	0.3037	0.2753	0.0000	1.0000
Religious tensions	0.2092	0.1873	0.0000	0.8889
Risk for exchange rate stability	0.0720	0.1222	0.0000	0.9250
Gdp pc (log)	8.9091	1.5357	5.6094	11.6260
Open	0.9430	0.6614	0.0017	4.4262
Inflation (%)	5.1898	8.5780	− 60.4964	152.561
Obs: 1494				

^5^ and

**Table 6:** List of countries

Angola	Latvia	Togo
Armenia	Lithuania	Trinidad and Tobago
Austria	Luxembourg	Tunisia
Bahamas	Madagascar	Turkey
Belarus	Malawi	Ukraine
Belgium	Malaysia	UK
Bolivia	Mali	USA
Brazil	Malta	Uruguay
Bulgaria	Mexico	Zambia
Burkina Faso	Moldova	
Canada	Mongolia	
Chile	Morocco	
China	Mozambique	
Colombia	Myanmar	
Congo (Republic)	Netherlands	
Costa Rica	New Zealand	
Croatia	Nicaragua	
Cyprus	Niger	
Denmark	Norway	
Dominican Republic	Oman	
Egypt	Panama	
Estonia	Paraguay	
Finland	Peru	
France	Philippines	
Gambia	Poland	
Germany	Portugal	
Greece	Qatar	
Guyana	Saudi Arabia	
Haiti	Sierra Leone	
Hong Kong	Singapore	
Hungary	Slovenia	
Iceland	South Africa	
India	Spain	
Ireland	Sri Lanka	
Israel	Sudan	
Italy	Tanzania	
Jordan	Thailand	

6.

## Footnotes

1 The panel unit root results are available upon request.

## Code availability

Code is available upon request.
