# Supplementary material for: Nonlinear nexus between corruption and tourism arrivals: a global analysis
Source: Empir Econ. 2022 Feb 7;63(4):1997–2024. doi: 10.1007/s00181-021-02193-2 (PMC8819199; doi:10.1007/s00181-021-02193-2)
Supplement: Supplementary file 1 — Supplementary file1 (DOCX 29 kb) [file 181_2021_2193_MOESM1_ESM.docx]

**Nonlinear nexus between corruption and tourism arrivals: a global analysis-**

**Online Appendix**

**Table 1. Threshold Tests and Threshold Estimates- Tourism Receipts**

| **Threshold variable** | **P-value** | **Threshold** | **n1** | **n2** |
| --- | --- | --- | --- | --- |
| Corruption (single threshold) | 0.0008 | 0.5000 | 606 | 744 |
| Corruption (double threshold) | 0.2629 | 0.5833 | - | - |
|  |  |  |  |  |

Table 1 presents the threshold test with the corresponding p-value and threshold estimate for tourism receipts for the null hypothesis of a linear model against the alternative of a threshold.

**Table 2. Threshold Regression-** **Tourism Receipts**

| **Variable** | **Linear**  **coef** | **Low Corruption**  **Regime**  $\boldsymbol{\leq}$**0.5000**  **coef** | **High Corruption**  **Regime**  $\boldsymbol{>}$ **0.5000**  **coef** |
| --- | --- | --- | --- |
| Corruption | -0.9622***  (0.1740) | 0.8604***  (0.2040) | -0.7008***  (0.2614) |
| Civil Disorder | 0.1672  (0.1047) | -0.2629  (0.1733) | 0.1347  (0.0999) |
| Civil War | -1.6132***  (0.1625) | -0.3636  (0.3727) | -1.3149***  (0.1017) |
| Economic Risk Rating | 0.0969  (0.1670) | 0.0630  (0.1331) | -0.8722***  (0.2733) |
| Ethnic Tensions | -1.4202***  (0.2915) | -0.9183***  (0.3279) | -0.9595***  (0.3539) |
| Financial Risk Rating | -0.3484  (0.2226) | -0.4641**  (0.2278) | -0.9045***  (0.2565) |
| Foreign Pressures | -1.1167***  (0.1416) | -0.3513  (0.2748) | -1.3046***  (0.1949) |
| Law & Order | 0.0862  (0.3112) | -0.1556  (0.4508) | -0.5268*  (0.2794) |
| Military in Politics | -0.7326**  (0.3147) | 0.0599  (0.4157) | -0.7632**  (0.3386) |
| Religious Tensions | -1.6548***  (0.3295) | -0.7957**  (0.3222) | -0.8118**  (0.3554) |
| Risk for Ex Rate Stability | -0.0569  (0.0910) | -0.0711  (0.0707) | -0.4617**  (0.1864) |
| GDP pc | 1.6777***  (0.1005) | 1.2730***  (0.0952) | 1.0950***  (0.0636) |
| Open | 0.2553***  (0.0584) | 0.2673***  (0.0658) | 0.1309*  (0.0676) |
| Inflation (%) | -0.0036**  (0.0018) | 0.0032  (0.0022) | -0.0130***  (0.0033) |

Table 2 presents the linear and the threshold regression model estimation results for tourism receipts. The second column includes the coefficient and robust standard errors (in parenthesis) for the linear panel fixed effects model. The remaining columns present the threshold regression estimation results (coefficients and robust standard errors). In all model estimations the variables are considered endogenous (including the threshold variable) and instrumented using their lag-values. All specifications always include an intercept and a time trend. Asterisks denote statistical significance at the 1% (***), 5% (**), and 10% (*) level.

**Table 3. Threshold Tests and Threshold Estimates- Tourist Arrivals and Control of Corruption Index**

| **Threshold variable** | **P-value** | **Threshold** | **n1** | **n2** |
| --- | --- | --- | --- | --- |
| Control of Corruption Index (single threshold) | 0.0033 | 0.2800 | 909 | 585 |
| Control of Corruption Index (double threshold) | 0.4513 | 0.5833 | - | - |
|  |  |  |  |  |

Table 3 presents the threshold test with the corresponding p-value and threshold estimate for tourist arrivals for the null hypothesis of a linear model against the alternative of a threshold using the control of corruption index.

**Table 4. Threshold Regression-Tourist Arrivals and Control of Corruption Index**

| **Variable** | **Linear**  **coef** | **High Corruption**  **Regime**  $\boldsymbol{\leq0.2800}$  **coef** | **Low Corruption**  **Regime**  $\boldsymbol{>}$ **0.2800**  **coef** |
| --- | --- | --- | --- |
| Control of Corruption Index | -0.3351***  (0.0648) | -0.3156***  (0.0407) | 0.0729*  (0.0397) |
| Civil Disorder | -0.2040***  (0.0750) | -0.2091**  (0.0983) | -0.1516  (0.1288) |
| Civil War | -0.2467***  (0.0916) | -0.2924**  (0.1226) | -0.1542***  (0.0250) |
| Economic Risk Rating | -0.0490  (0.1374) | -0.0087  (0.1931) | -0.2980  (0.2231) |
| Ethnic Tensions | -0.4015***  (0.1299) | -0.5789***  (0.1558) | -0.0788  (0.2578) |
| Financial Risk Rating | -1.1455***  (0.1315) | -1.1344***  (0.1682) | -0.3660***  (0.1244) |
| Foreign Pressures | -0.3504***  (0.0752) | -0.6662***  (0.1070) | -0.0846  (0.1143) |
| Law & Order | -0.1108  (0.1517) | -0.1638  (0.1834) | -0.2381  (0.2654) |
| Military in Politics | -0.1413  (0.1193) | -0.3562**  (0.1539) | -0.3501*  (0.1814) |
| Religious Tensions | -0.0728  (0.1256) | -0.1047  (0.1625) | -0.3353  (0.2142) |
| Risk for Exchange Rate Stability | -0.2518***  (0.0726) | -0.1426  (0.0996) | -0.1672  (0.1200) |
| GDP pc | 1.2053***  (0.1648) | 1.0650***  (0.0553) | 1.1132***  (0.0667) |
| Open | 0.0027***  (0.0005) | 0.0017***  (0.0006) | 0.0408***  (0.0082) |
| Inflation (%) | -0.0020  (0.0014) | -0.0021***  (0.0005) | -0.0031  (0.0026) |

Table 4 presents the linear and the threshold regression model estimation results for tourist arrivals and the control corruption index. The second column includes the coefficient and robust standard errors (in parenthesis) for the linear panel fixed effects model. The remaining columns present the threshold regression estimation results (coefficients and robust standard errors). In all model estimations the variables are considered endogenous (including the threshold variable) and instrumented using their lag-values. All specifications always include an intercept and a time trend. Asterisks denote statistical significance at the 1% (***), 5% (**), and 10% (*) level.

**Table 5. BMA results for the linear and threshold model for Tourist Arrivals**

|  | **BMA Linear Model** | | **BMA Threshold Model** | | | |
| --- | --- | --- | --- | --- | --- | --- |
| **Variable** | **pip** | **pm/(pse)** | **pip** | **Low Corruption Regime**  $\boldsymbol{\leq}$**0.5208**  **pm/(pse)** | **pip** | **High Corruption Regime**  $\boldsymbol{>}$ **0.5208**  **pm/(pse)** |
| Corruption | 1.00 | -0.7396***  (0.2802) | 1.00 | 1.6583***  (0.4156) | 1.00 | -1.7801***  (0.6743) |
| Civil Disorder | 0.53 | -1.2371  (0.8901) | 0.53 | -0.2921  (0.5373) | 0.26 | -0.2135  (0.4082) |
| Civil War | 1.00 | -1.4330***  (0.2941) | 0.55 | -0.6468  (0.9861) | 0.82 | -0.9158*  (0.5371) |
| Economic Risk Rating | 0.12 | -0.1122  (0.3570) | 0.04 | -0.0058  (0.1499) | 0.83 | -0.9347*  (0.5658) |
| Ethnic Tensions | 1.00 | -0.8785***  (0.0969) | 0.05 | -0.0111  (0.0762) | 0.92 | -0.7363**  (0.3349) |
| Financial Risk Rating | 1.00 | -3.3777***  (0.3966) | 1.00 | -0.9568***  (0.2490) | 1.00 | -1.0675***  (0.3232) |
| Foreign Pressures | 0.03 | -0.0061  (0.0533) | 0.17 | -0.1047  (0.2750) | 1.00 | -0.1196***  (0.0354) |
| Law & Order | 0.40 | -0.2532  (0.3444) | 0.11 | -0.0680  (0.2363) | 0.43 | -0.3310  (0.4285) |
| Military in Politics | 1.00 | -1.1038***  (0.2022) | 0.07 | -0.0225  (0.1225) | 1.00 | -1.2607***  (0.2676) |
| Religious Tensions | 1.00 | -1.3063***  (0.1772) | 1.00 | -0.9074***  (0.3192) | 1.00 | -0.9869***  (0.2284) |
| Risk for Exchange Rate Stability | 0.24 | -0.1839  (0.3735) | 0.04 | -0.0021  (0.1015) | 0.91 | -0.3896**  (0.1918) |
| GDP pc | 1.00 | 0.6613***  (0.0397) | 1.00 | 0.7778***  (0.0648) | 1.00 | 0.6465***  (0.0571) |
| Open | 1.00 | 0.1556***  (0.0554) | 1.00 | 0.4049***  (0.0556) | 1.00 | 0.1212***  (0.0127) |
| Inflation (%) | 1.00 | -0.0031***  (0.0006) | 0.26 | -0.0085  (0.0164) | 0.18 | -0.0021  (0.0051) |

Table 5 presents BMA results for the linear and threshold model for Tourist Arrivals. The posterior inclusion probability (PIP), is the sum of posterior model probabilities over all those models that contain that variable. The posterior mean (PM) is the average of the coefficient estimates (COEF) of individual models weighted by the posterior model probability. The posterior standard error (PSE) is the BMA estimate for the standard error (SE). All specifications always include an intercept and a time trend. Asterisks denote statistical significance at the 1% (***), 5% (**), and 10% (*) level.
